# Comparison Between Hesperidin, Coumarin, and Deferoxamine Iron Chelation and Antioxidant Activity Against Excessive Iron in the Iron Overloaded Mice

**DOI:** 10.3389/fnins.2021.811080

**Published:** 2022-02-01

**Authors:** Mahdi Aalikhani, Yagoub Safdari, Mehrdad Jahanshahi, Mehrdad Alikhani, Masoumeh Khalili

**Affiliations:** ^1^Student Research Committee, Golestan University of Medical Sciences, Gorgan, Iran; ^2^Department of Medical Biotechnology, School of Advanced Technologies in Medicine, Golestan University of Medical Sciences, Gorgan, Iran; ^3^Golestan Research Center of Gastroenterology and Hepatology, Golestan University of Medical Sciences, Gorgan, Iran; ^4^Neuroscience Research Center, Department of Anatomy, Faculty of Medicine, Golestan University of Medical Sciences, Gorgan, Iran; ^5^Department of Cardiology, Shahid Beheshti University of Medical Sciences, Tehran, Iran; ^6^Neuroscience Research Center, Golestan University of Medical Sciences, Gorgan, Iran; ^7^Infectious Diseases Research Center, Golestan University of Medical Sciences, Gorgan, Iran

**Keywords:** antioxidant activity, Alzheimer’s diseases, brain, free radicals, antioxidant enzymes, iron chelating agent, hesperidin, coumarin

## Abstract

**Objective:**

Iron accumulation in the brain leads to the development of Alzheimer’s and Parkinson’s diseases. Nowadays, iron chelation therapy is the best way to decrease the side effects of iron and amyloid plaques accumulation. Iron chelators are commonly used for the treatment of Alzheimer’s disease. Previous studies have shown that natural products such as phenol and flavonoid compounds could chelate heavy metals. In the current study, we examined the iron chelation activity of hesperidin and coumarin on the brain tissue of iron-overloaded mice.

**Methods:**

48 NMRI male mice were divided into eight groups (*n* = 6). Six groups were treated with iron dextran (100 mg/kg/day) four times a week for 6 weeks. After stopping the injections for a month, five groups of iron-overloaded mice were treated with hesperidin, coumarin, and desferal four times a week subsequent for four subsequent weeks. Finally, the mice were anesthetized, and blood samples were collected from the ventricle of the heart for subsequent examination. The brain tissues were isolated and fixed in the 4% paraformaldehyde solution for Perl’s staining.

**Results:**

The results show that hesperidin and coumarin could strongly chelate excessive iron from the serum and deposit iron from the brain tissue compared to desferal group. Catalase and super oxidase activity were decreased in the iron-overloaded group, but in the treated group by hesperidin and coumarin, the enzyme’s activity was increased significantly.

**Conclusion:**

Hesperidin and coumarin, as natural products, are powerful options to chelate iron ions and increase the activity of antioxidant enzymes.

## Introduction

Iron is one of the most important transition metals in the living system, which is necessary for metabolism. It works in redox-active enzymes and oxygen transportation (Baccan et al., 2012). The accumulation of excessive Fe cation generates oxidative stress. Some peptides such as ferritin, heme protein, and transferrin stabilize iron in its trivalent state. This protects from the production and generation of harmful reactive oxygen species (ROS) such as superoxide anion (O2^–^), hydrogen peroxide (H_2_O_2_), and hydroxyl radicals (.OH). Iron overload leads to Fenton reaction and Haber–Weiss reactions, which cause numerous damages to proteins, membranes, and DNA (Piloni et al., 2013; Khalili et al., 2015a). Cells could control ROS by antioxidant enzymes such as superoxide dismutase (SOD), catalase (CAT), and peroxidase (POD) (Mokni et al., 2007). Extra Fe leads to the increased labile plasma iron, which is related to neurological damages after ischemia, endothelial dysfunction among thalassemic children, cognitive deficits in Alzheimer’s patients, Parkinson’s and Huntington’s diseases, complications of myelodysplastic syndrome, especially infection and tissue damage, and diabetes (Baccan et al., 2012; Piloni et al., 2013; Khalili et al., 2015a). Fe could cross the blood-brain barrier (Zheng and Monnot, 2012). There are two ways to produce the iron overloaded animal for animal study: 1- orally diet, and 2- intraperitoneal injection (i.p.) (Piloni and Puntarulo, 2010). Our previous studies showed that *i.p.* injection of iron-dextran could lead to iron deposition in the liver, brain, and plasmas of the mice (Khalili et al., 2015a,b; Ebrahimzadeh et al., 2016). Additionally, naringin, as a natural product and an iron chelation agent, could chelate iron from the brain and serum of the iron-overloaded mice (Jahanshahi et al., 2021).

This effect observed to be accompanied by the enhanced SOD and CAT activities, suggesting that chronic Fe administration may have induced the adaptive responses involved in the stimulation of the antioxidant defense systems. The underlying mechanisms of tissue damage are not clear yet, but they may probably depend on the Fe administration’s protocol. Although lipid damage was observed in many cases after Fe overload, antioxidant capacity appeared to play a crucial role in controlling the impairment mechanisms.

Nowadays, the morbidity of AD is increasing. Therefore, it is important to find new drugs for the treatment of AD. Previous studies have shown that some natural products such as hesperidin (PubChem CID: 10621) and coumarin (PubChem CID: 323) have antioxidant and iron chelation activities (Grazul and Budzisz, 2009; Wang et al., 2014). Hesperidin is a flavanone glycoside found in citrus fruits, and coumarin is a phenolic substance composed of the fused benzene and α-pyrone rings, which can be found in citrus fruits and carrots (Bor et al., 2016; Rasouli et al., 2019). Both Hesperidin and coumarin can cross the brain–blood barrier (BBB) and have neuroprotective activity (Grazul and Budzisz, 2009; Golechha et al., 2011; Thenmozhi et al., 2015; Jabbar et al., 2016). Therefore, in this study, we examined the iron chelation activity of both hesperidin and coumarin on iron-overloaded mice.

## Materials and Methods

### Assessment of Metal Chelating Activity

To assess the metal chelating activity of hesperidin and coumarin, 1 ml of each fraction (800 μg mL^–1^) was added to 0.5 mL of 2 mM FeCl_2_ solution. Thereafter, the reaction was started by adding 5 mM ferrozine, and the absorbance of the solution was read using a spectrophotometer at 562 nm. EDTA was used as the standard (Khalili and Ebrahimzadeh, 2015).

### Animals and Injection Protocol

Forty-eight NMRI albino male mice [that was developed by TACONIC (2021), aged 6–7 weeks, weighted 20–25 g] were placed in cases, as six rats per cage, and then maintained at a light cycle of 12 h/light 12 h dark under the controlled temperature and humidity (24 ± 2°C and 45–55%, respectively). All the animals were obtained from by Pasteur Institute (Amol, Northern Iran). We performed all the animal experiments in terms of the ethical guidelines approved by the Ethical Committee of Golestan University of Medical Sciences, Gorgan, Iran (approval number: ir.goums.rec.1396.40). The mice were classified into eight groups, each one consisting of six mice. Then subjected to different treatments after inducing iron-overload condition by *i.p.* injection of iron dextran (100 mg/kg/day) four times per week for a duration of four subsequent weeks. After that, the animals did not receive any treatments for a month. Then mice were classified in different groups and treated as described in Table 1; Khalili et al. (2015b); Sripetchwandee et al. (2016).

**TABLE 1:** Classification of animal groups.

Groups	Explanation of the animals groups	First treatment (6 weeks and 4 times in the weeks) (*i.p* injection)	Second treatment (4 weeks no any injection)	Third treatment (4 weeks and 4 times in the weeks) (*i.p* injection)
Group A	Control group: Normal saline-receiving group	Normal saline	No injection	Normal saline
Group B	normal saline and deferoxamine-receiving group	Normal saline	No injection	25 mg/kg/day deferoxamine
Group C	Iron dextran and normal saline-receiving group	100 mg/kg/day Iron dextran	No injection	Normal saline
Group D	Iron dextran and Deferoxamine-receiving group	100 mg/kg/day Iron dextran	No injection	25 mg/kg/day deferoxamine
Group E	Low dose hesperidin-receiving group	100 mg/kg/day Iron dextran	No injection	50 mg/kg/day hesperidin
Group F	High dose hesperidin-receiving group	100 mg/kg/day Iron dextran	No injection	100 mg/kg/day hesperidin
Group G	Low dose coumarin-receiving group	100 mg/kg/day Iron dextran	No injection	50 mg/kg/day coumarin
Group H	High dose coumarin-receiving group	100 mg/kg/day Iron dextran	No injection	100 mg/kg/day coumarin

Then, the mice were anesthetized with ketamine (90 mg/kg, *i.p.*) and xylazine (10 mg/kg, *i.p.*). Blood samples were directly obtained from the mice’s heart ventricles. Afterward, the mice’s blood serums were isolated and stored at −20°C. In order to determine the total iron content, CAT activity and SOD activity, three brain tissues were kept in the PBS solution at −70°C. The other brain tissues were kept in 4% paraformaldehyde solution for Perl’s staining.

### Assessment of the Non-heme Iron Content of Serum and Brain Samples

Brain’s non-heme iron content was measured using the method described by Rebouche et al. (2004). Briefly, 30 μl of brain tissue was homogenized in the 1:10 (w/v) in high-purity water, mixed with 1 N HCl and 10% trichloroacetic acid. Samples were incubated in a Bain-marie for 1 h at 95°C. Subsequently, the mixture was centrifuged at 8,200 × *g* for 10 min. In addition, 30 μl of the supernatant was blended with a solution containing 1.5% (v/v) thioglycolic acid, 1.5 mol/l sodium acetate, and 0.508 mmol/l ferrozine that were prepared in high-purity water and incubated for 30 min at room temperature. The absorbance rate was evaluated at 562 nm using a spectrophotometer (Unico 2100 Vis spectrophotometer, NJ, United States). As well, the blank one contained a mixture of 30 μl high-purity water and 1.5 mol/l sodium acetate containing 0.1 or 1.5% thioglycolic acid. The iron standard was daily prepared and 0, 2, 4, 6, 8, and 10 μg/ml of the iron standard solution was mixed with an equal volume of 1 N HCl and 10% trichloroacetic acid in high-purity water.

### Assay of Catalase Activity in the Brain Tissues

At this stage, we used a CAT kit (ZellBio GmbH, Germany) to evaluate catalase activity in the brain tissues of the treated mice. Briefly, for this purpose, 100 mg of the brain tissues were homogenized in 1 ml PBS (100 mM, pH 7.4) using a Dounce homogenizer. Thereafter, it was centrifuged at 6,000 RPM for 20 min. The supernatants were collected and further steps were performed according to the CAT kit. Finally, the absorbance of the samples were read using a microplate reader/ELISA at 405 nm.

### Assay of Superoxide Dismutase Activity in the Brain Tissues

For the assessment of superoxide dismutase activity in the brain tissues of mice, we used a SOD kit (ZellBio GmbH, Germany). In brief, for this purpose, 100 mg of the brain tissues were homogenized in 1 ml of PBS (by Dounce homogenizer). Afterward, it was centrifuged at 5,000 rpm for 20 min. Thereafter, 10 μl of the supernatant was added to 250 μl of solution R1, 10 μl of solution R2, 10 μl dd-water, and 20 μl chromogen. The blank sample contained 10 μl dd-water, 250 μl of solution R1, and 30 μl dd-water. All the reagents were mixed well, and then the absorbance was read at times 0 and 2 min using the ELISA reader at 420 nm.

### Brain Histology

The brain tissues were fixed in the paraformaldehyde (4%). All the removed brain tissues were processed using an automated tissue processing machine (Did Sabz, Urmia, Iran) and then embedded in paraffin for performing a histological analysis (Ebrahimzadeh et al., 2016; Jahanshahi et al., 2020). The brain sections (6 μm) were taken using a rotary microtome (Pooyan MK 1110, Iran).

Perl’s Stain kit (Shimi Pajhohesh Asia, Amol, Iran) was used for the whole brain staining. Briefly, the brain section was deparaffinized, hydrated, and then placed in potassium ferrocyanide solution. Afterward, it was put in ferrocyanide-hydrochloric acid solution. The tissues were rinsed with distilled water and counterstained with a nuclear fast red solution after performing another round of rinsing. Next, they were dehydrated in 95% alcohol, as absolute alcohol, and cleared in xylene. Finally, the tissues were cover-slipped with Entellan glue (Merck, Germany). As well, the needed pictures were taken using a light microscope (Model: BX 53, Olympus, Japan) at a 40× magnification equipped *via* a digital camera (Model: DP73, Olympus, Japan) (Khalili et al., 2015a,b).

### Statistical Analysis

Variance analysis (one-way ANOVA) was carried out on the obtained data using Graph Pad Prism 5 (Means were compared using Newman–Keuls multiple comparison tests). Statistical significance was set at *P* < 0.05. As well, means were reported ± SD.

## Results

### *In vitro* Iron Chelating Activity of Hesperidin and Coumarin

Iron chelation activity of hesperidin and coumarin was measured under the *in vitro* condition. The results showed that the iron chelation activities of hesperidin and coumarin (concentration 50 μg/ml) were 58 and 50%, respectively. The iron chelation activity of EDTA (10 μg/ml) as the standard was 93%.

### Non-heme Iron Content in Serum and Brain

**FIGURE 1:**
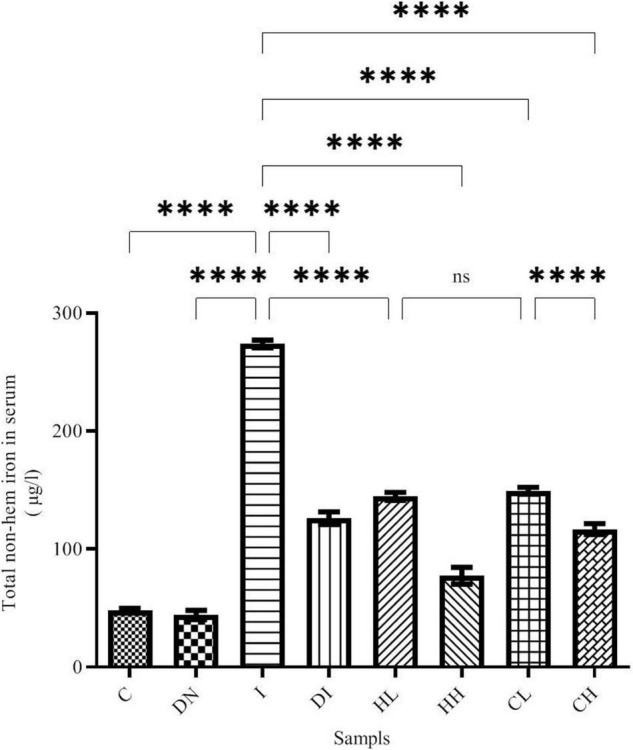
Serum non-heme iron content in the treated mice, Group C, control group: Normal saline-receiving group; Group DN, normal saline and deferoxamine-receiving group; Group I, iron dextran and normal saline-receiving group; Group DI, iron dextran and deferoxamine-receiving group; Group HL, low dose hesperidin-receiving group; Group HH, high dose hesperidin-receiving group; Group CL, low dose coumarin-receiving group; and Group CH, high dose coumarin-receiving group (Mean ± SD; ^****^p ≤ 0.0001, ns: non-significant).

Non-heme iron content was assessed using ferrozine methods. As shown in Figure 1, total non-heme iron content changed in the blood serum after the treatment. The maximum and minimum iron contents in the serum were obtained in group I (iron-overloaded group) and group C (273.9 ± 3.32 μmol/L and 47.87 ± 1.89 μmol/L, respectively, *p* < 0.001). The non-heme iron content in the group ND (44.16 ± 3.85 μmol/L) decreased compared to the group C; however, there was no significant difference between the groups’ ND and C. Non-heme iron content decreased in the Group DI (126.3 ± 5.26 μmol/L). Moreover, a significant difference was found between the groups I and DI (*p* < 0.001). Iron content decreased in the treated mice by low and high doses of hesperidin (144.7 ± 3.35 and 77.52 ± 7.06 μmol/L, respectively). Of note, there was a significant difference among groups HL, HH, and the group I (*p* < 0.001). The highest iron depletion was found to be related to the group CH (coumarin 100 mg/kg/day, *p* < 0.001). The results showed that the non-heme iron content decreased in the groups’ CL and CH. In this regard, there were significant differences between group CL and CH and group I (*p* < 0.001) (Figure 1).

**FIGURE 2:**
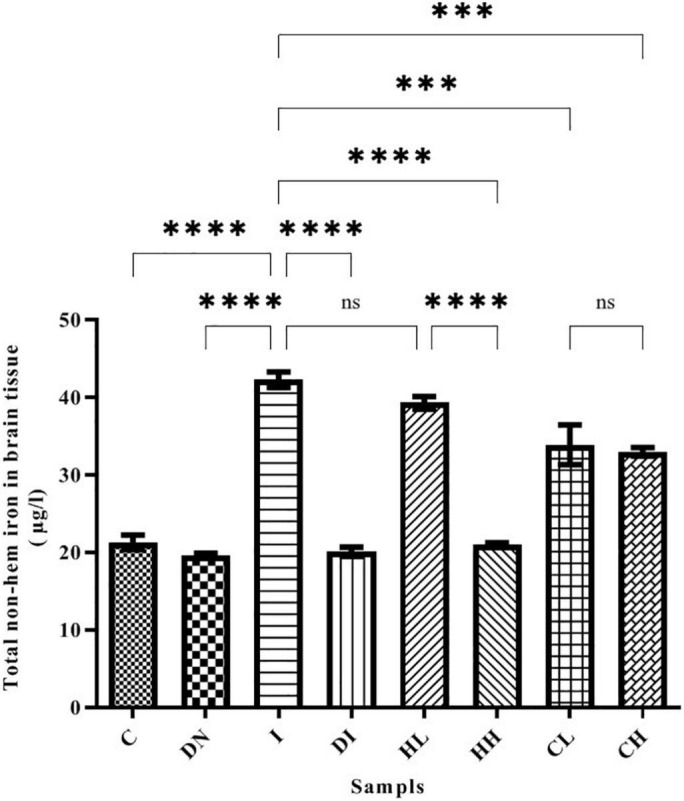
Brain tissue’s non-heme iron content in the treated mice, Group C, control group: Normal saline-receiving group; Group DN, normal saline and deferoxamine-receiving group; Group I, iron dextran and normal saline-receiving group; Group DI, iron dextran and deferoxamine-receiving group; Group HL, low dose hesperidin-receiving group; Group HH, high dose hesperidin-receiving group; Group CL, low dose coumarin-receiving group; and Group CH, high dose coumarin-receiving group (Mean ± SD; ^***^p ≤ 0.001, ^****^p ≤ 0.0001, ns: non-significant).

Our results show that the non-heme iron content in the brain increased up to 42.27 ± 0.99 μg/l in group I, while in group C (control group treated by normal saline), this was 21.28 ± 0.93 μg/l (Figure 2). There was a significant difference between groups C and I (*p* < 0.001). Non-heme iron content significantly decreased in the mice treated by desferal and natural compounds. As shown by the results, the maximum decrease of non-heme iron content was obtained in the group DI (20.10 ± 0.59 μg/l) and there was a significant difference with the group I (*p* < 0.001). In the group HH treated by hesperidin 100 mg/kg/day, non-heme iron content in the mice’s brain decreased (21.05 ± 0.16 μg/l), and there was no significant difference with the group DI. Additionally, Non-heme iron content decreased in the groups’ CL and CH treated with 50 and 100 mg/kg/day of coumarin (33.87 ± 2.56 and 32.98 ± 0.53 μg/l, respectively) (Figure 2).

### Superoxide Dismutase Activity in Serum and Brain Tissues

**FIGURE 3:**
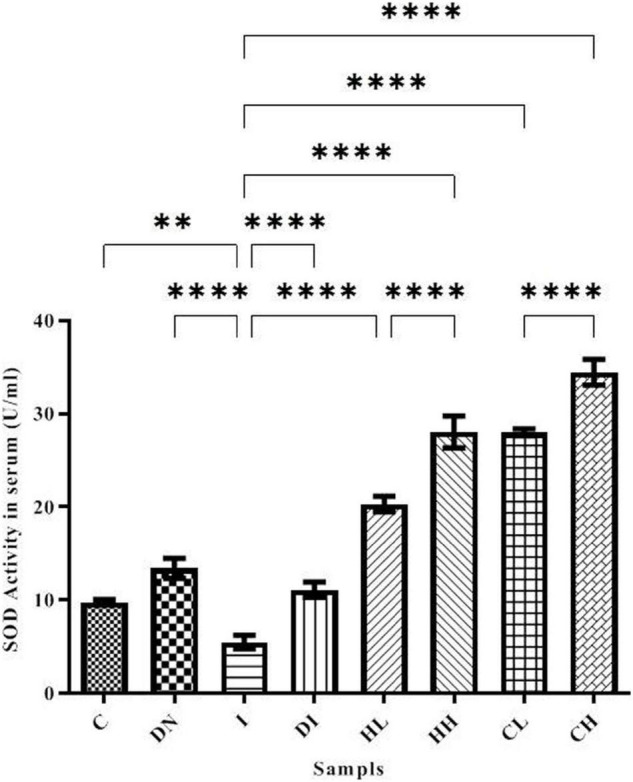
SOD activity in the serum of the treated mice, Group C, control group: Normal saline-receiving group; Group DN, normal saline and deferoxamine-receiving group; Group I, iron dextran and normal saline-receiving group; Group DI, iron dextran and deferoxamine-receiving group; Group HL, low dose hesperidin-receiving group; Group HH, high dose hesperidin-receiving group; Group CL, low dose coumarin-receiving group; and Group CH, high dose coumarin-receiving group (Mean ± SD; ^**^p ≤ 0.01, ^****^p ≤ 0.0001).

Superoxide dismutase activity in serum is shown in Figure 3. The minimum activity of SOD was obtained in the group I (5.49 ± 0.72 U/ml). SOD activity in the C and DN groups was 9.74 ± 0.31 and 13.39 ± 1.03 U/ml, respectively. In the group DI, SOD activity increased dramatically. Notably, there were significant differences between groups DI and I (*p* < 0.001). The treatment by natural products caused increased SOD activity. The highest SOD activity was obtained in the CH group (34.47 ± 1.47 U/ml) (Figure 3).

**FIGURE 4:**
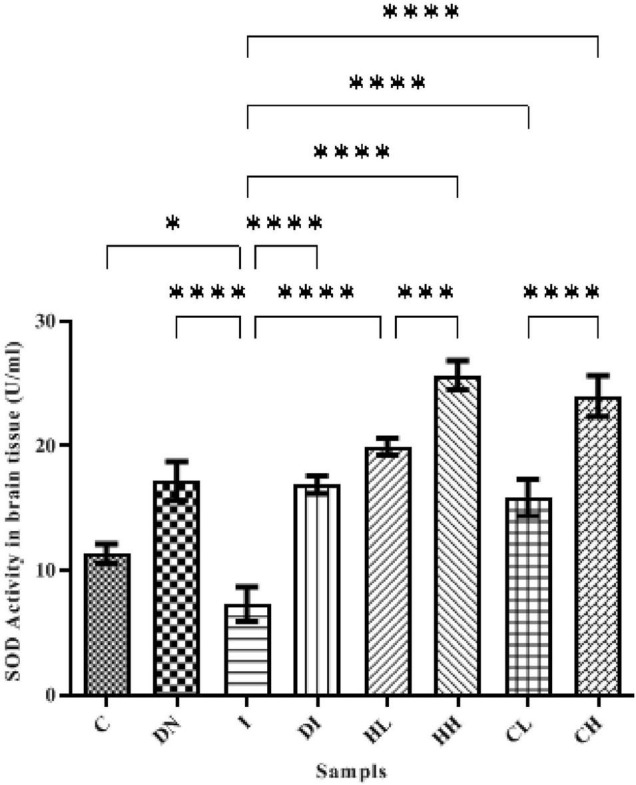
SOD activity in the brain tissue of the treated mice, Group C, control group: Normal saline-receiving group; Group DN, normal saline and deferoxamine-receiving group; Group I, iron dextran and normal saline-receiving group; Group DI, iron dextran and deferoxamine-receiving group; Group HL, low dose hesperidin-receiving group; Group HH, high dose hesperidin-receiving group; Group CL, low dose coumarin-receiving group; and Group CH, high dose coumarin-receiving group (Mean ± SD; *p ≤ 0.05, ^***^p ≤ 0.001, ^****^p ≤ 0.0001).

SOD activity in the brain of the controls was assessed as 11.35 ± 0.78 U/ml. In the group DN, SOD activity in the brain increased up to 17.17 ± 1.51 U/ml, and there were significant differences between groups C and DN (*p* < 0.001). The minimum activity of SOD was obtained in the serum of group I (7.28 ± 1.37 U/ml), there were significant differences between groups C and I (*p* < 0.0001). Following the treatment by DFO, hesperidin, and coumarin, SOD activity increased. The highest activity of SOD was obtained in the group’s HH (25.66 ± 1.27 U/ml) and CH (23.99 ± 1.63 U/ml). SOD activity in the group DI also increased up to 16.78 ± 0.71 U/ml (Figure 4).

### Catalase Activity in Serum and Brain Tissues

**FIGURE 5:**
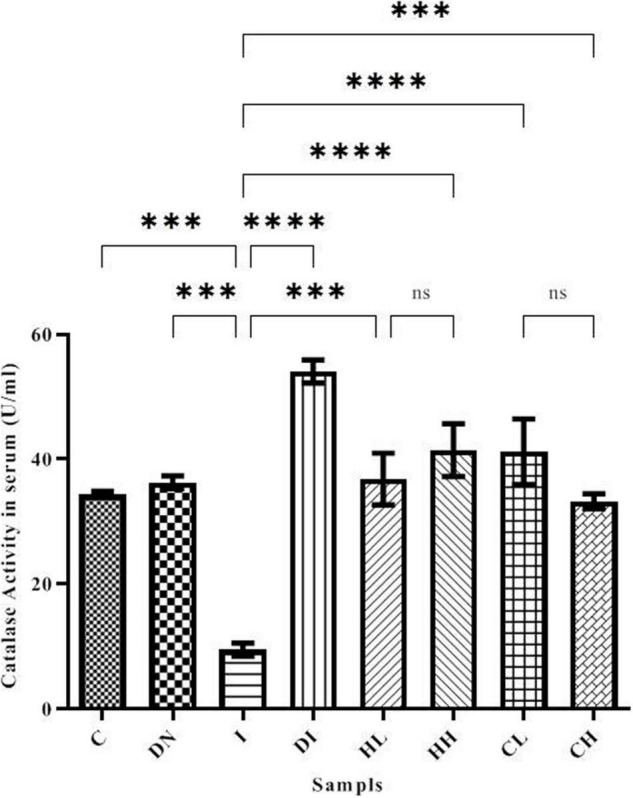
CAT activity in the serum of the treated mice, Group C, control group: Normal saline-receiving group; Group DN, normal saline and deferoxamine-receiving group; Group I, iron dextran and normal saline-receiving group; Group DI, iron dextran and deferoxamine-receiving group; Group HL, low dose hesperidin-receiving group; Group HH, high dose hesperidin-receiving group; Group CL, low dose coumarin-receiving group; and Group CH, high dose coumarin-receiving group (Mean ± SD; ^***^p ≤ 0.001, ^****^p ≤ 0.0001, ns: non-significant).

Catalase activity in the serum is shown in Figure 5. Serum CAT activity in groups C and DN was estimated as 34.37 ± 0.50 and 36.29 ± 1.02 U/ml, respectively. The minimum CAT activity was found in the group I (9.49 ± 1.02 U/ml). As well, the maximum CAT activity was obtained in the group DI (54.08 ± 1.86 U/ml). The treatment by hesperidin and coumarin caused the increased level of CAT activity in the serum. CAT activities in the mice treated with low and high doses of hesperidin was 36.83 ± 4.16 and 41.46 ± 5.26 U/ml, respectively. The CAT activity in the serum of the CL and CH groups was obtained as 41.22 ± 5.26 and 33.29 ± 1.19 U/ml, respectively.

**FIGURE 6:**
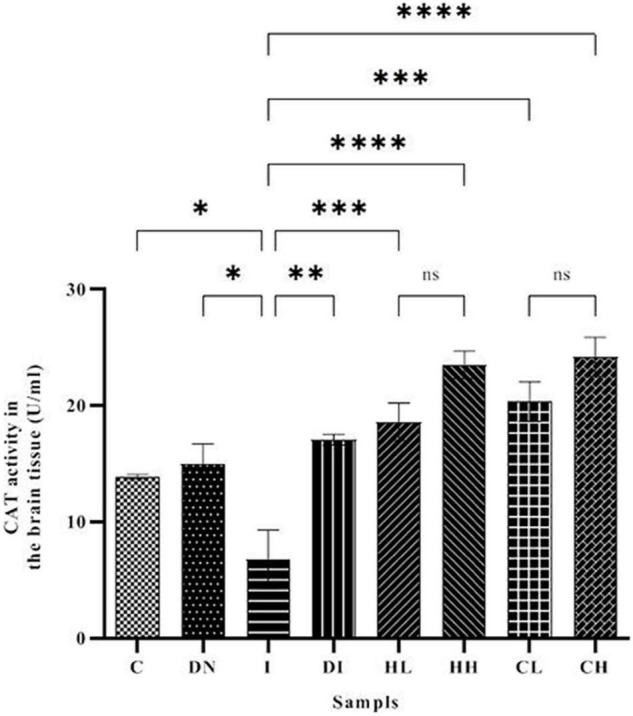
CAT activity in the brain tissue of the treated mice, Group C, control group: Normal saline-receiving group; Group DN, normal saline and deferoxamine-receiving group; Group I, iron dextran and normal saline-receiving group; Group DI, iron dextran and deferoxamine-receiving group; Group HL, low dose hesperidin-receiving group; Group HH, high dose hesperidin-receiving group; Group CL, low dose coumarin-receiving group; and Group CH, high dose coumarin-receiving group (Mean ± SD; *p ≤ 0.05, ^**^p ≤ 0.01, ^***^p ≤ 0.001, ^****^p ≤ 0.0001, ns: non-significant).

As shown in Figure 6, the CAT activity in the brain tissues of the mice increased up to 23.49 ± 1.18 and 24.18 ± 1.69 U/ml in the HH and CH groups, respectively. The minimum CAT activity of the brain was obtained in the group I (6.79 ± 2.51 U/ml). The CAT activity in the control group was 13.87 ± 0.19 U/ml. In addition, CAT activity in the iron-overloaded mice treated by DFO increased to 17.08 ± 0.45 U/ml.

### Histology

#### Perl’s Staining of the Mice’s Brain Tissue

**FIGURE 7:**
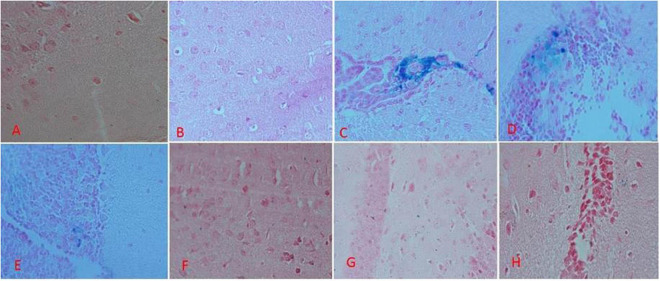
Brain tissue perl’s staining in the treatment mice. **(A)** Control group: Normal saline-receiving group. **(B)** Normal saline and deferoxamine-receiving group. **(C)** Iron dextran and normal saline-receiving group. **(D)** Iron dextran and deferoxamine-receiving group. **(E)** Low dose hesperidin-receiving group. **(F)** High dose hesperidin-receiving group. **(G)** Low dose coumarin-receiving group. **(H)** High dose coumarin-receiving group.

The results of Perl’s staining of the brain tissues is shown in Figure 7. Accordingly, the blue spots are the iron depositions. As shown in Figures 7A,B, regarding the mice in the group A and B, there was no iron deposition. In the group C treated with iron dextran, there were countless iron depositions (Figure 7C). In the other groups, which were iron-overloaded and treated with DFO and natural compounds, the iron deposition decreased in the brain (Figures 7E–H).

## Discussion

Iron-overloaded animals are one way to study diseases related to the iron accumulation (Khalili et al., 2015a; Ebrahimzadeh et al., 2016; Jahanshahi et al., 2021). Using the metal chelation agents is an important way to decrease the iron-overload’s side effect (Jahanshahi et al., 2021). Hesperidin is a flavonoid, which can be found in the *C. sinensis Osbeck*, *C. limon Burm. f.*, and *C. grandis Osbeck* (Zou et al., 2016). Coumarin is a phenolic compound rich in citrus fruits (Zou et al., 2016). Both Hesperidin and coumarin have antioxidant activity and can decrease free radical production (Lin et al., 2008; Nakao et al., 2011). Some natural products have metal chelation and antioxidant activities such as citrus fruits, hesperidin, coumarin, naringin, coumarin, resveratrol, quercetin, naringin, and silymarin (Mokni et al., 2007; Cui et al., 2014; Dong et al., 2014; Wang et al., 2014; Ebrahimzadeh et al., 2015, 2016; Filipsky et al., 2015; Khalili et al., 2015a; Zou et al., 2016; Chen et al., 2018; Jahanshahi et al., 2021). Our results show that hesperidin and coumarin have potential iron chelation activities. Therefore, these could decrease iron in the brain tissue and serum of the iron-overloaded mice (Figures 1, 2). Hesperidin and coumarin, as natural products, have antioxidant and anti-inflammatory activities and could inhibit free radicals (Wilmsen et al., 2005; Kadhum et al., 2011; Bubols et al., 2013; Witaicenis et al., 2014; Parhiz et al., 2015; Cao et al., 2018). The maximum decrease of the total iron content in the serum was obtained by a high dose of hesperidin. Hesperidin and coumarin were observed to have more potential iron chelation activities compared to DFO. Our results show that the iron chelation activity of hesperidin and coumarin in the serum was in a dose-dependent manner. Lu and Serrero in their study showed that resveratrol inhibits the growth of human breast cancer cells in a dose-dependent manner (Lu and Serrero, 1999). Tocotrienol-rich fraction, as a natural product, had some dose-dependent effects on serum total cholesterol and LDL-cholesterol (Qureshi et al., 2002). Our previous study demonstrated that naringin could chelate excessive iron from the brain tissues and serum of the iron-overloaded mice (Jahanshahi et al., 2021). Iron accumulation in the brain occurs with aging. Accumulation of iron in the brain eventually leads to the development of some neuro-cerebral diseases such as Alzheimer’s and Parkinson’s (Masaldan et al., 2019). A previously performed case report reported that iron chelating treatment with deferiprone in a 61-year-old woman with symptoms of neurodegeneration and brain iron accumulation for 6 months resulted in the patient’s gait improvement (Forni et al., 2008). Some studies have previously shown that natural products prevent the brain from producing heavy metals and free radicals’ side effects (La Grange et al., 1999; Maynard et al., 2002; Gu et al., 2016; Jahanshahi et al., 2021). Excessive iron leads to Fenton reaction and then produce free radicals like ROS. Correspondingly, ROS damages biological molecules and leads to different diseases; therefore, using the iron-chelating agent is a fast and effective therapeutic way to decrease the dangers of iron accumulation (Badria et al., 2015; Khalili et al., 2015a,b; Jahanshahi et al., 2021).

**FIGURE 8:**
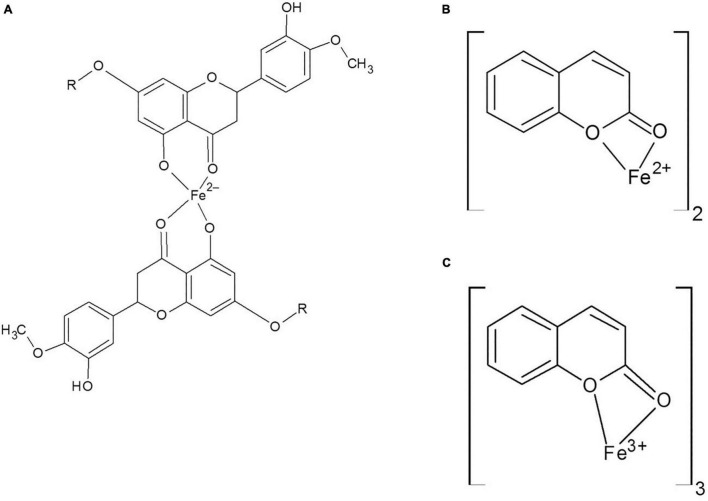
**(A)** Hesperidin and Fe binding site, **(B,C)** Coumarin and Fe^2 +^ and Fe^3 +^ binding.

It was indicated that coumarin and hesperidin could protect the brain from oxidative stress and have neuroprotective effect as well (Grazul and Budzisz, 2009; Khan and Parvez, 2015; Thenmozhi et al., 2015). As shown in Figure 2, brain total iron content decreased by the use of natural products, especially HH. There were no significant differences among the C, ND, DI, and HH groups. Our results show that SOD and CAT activities decreased in the iron-overloaded group. As previous studies have shown, increasing the concentration of iron suppresses and reduces the activity of the enzyme catalase. This could be due to the destruction of the “heme” group in the catalase protein by iron peroxidation. Therefore, with the improvement of antioxidant conditions, the activity of catalase improves (Levin et al., 1973; Galleano and Puntarulo, 1997; Zhao et al., 2005). In the groups treated with DFO, hesperidin, and coumarin, the activity of antioxidant enzymes increased dramatically. CH caused increased SOD activity in both the brain and serum. The highest brain SOD activity was obtained in the HH and CH groups. Of note, hesperidin and coumarin stimulated the activity of the SOD enzyme more than DFO, indicating their greater antioxidant power. As well, serum CAT activity dramatically increased in the hesperidin and coumarin groups, but DFO was more potent compared to the others. Brain CAT activity increased in the treatment group by administrating the natural products. The results show that hesperidin and coumarin antioxidant activity is in a dose-dependent manner. Zou et al. (2016) in their study showed that citrus flavonoids such as hesperidin, rutin, quercetin, naringenin, and naringin have different antioxidant activities. They also showed that citrus flavonoids could improve the oxidant enzymes and antioxidant activity under both *in vivo* and *in vitro* conditions (Zou et al., 2016). Citrus flavonoids could scavenge ROS and free radicals, anti-lipid oxidation activity *in vitro* and *in vivo*, and decrease the quality of peroxidation formation under *in vivo* conditions (Zou et al., 2016; Jahanshahi et al., 2021). Citrus phenolic acid-like coumarins could also scavenge free radicals because they have antioxidant activity considering their phenolic hydroxyl group in molecule structure. As well, coumarins by inhibiting XO, decrease the cellular free radical production (Zou et al., 2016). The results of brain tissues‘ Prussian blue staining showed that iron sedimentation decreased after the treatment with both hesperidin and coumarin. In this regard, brain tissues’ Prussian blue staining shows that hesperidin and coumarin chelated more iron than DFO. It is noteworthy that iron accumulation is found in the brain, cerebellum, parts of the hippocampus, nucleus accumbens, and substantia nigra (Masaldan et al., 2019). The results of brain tissue Prussian blue staining showed that iron was accumulated in the iron-overload group, and iron deposition was observed in the cerebral cortex, cerebellum, and hippocampus. The treatment with natural products reduced iron accumulation, oxidative stress, and ROS production from the brain tissues (Mokni et al., 2007; Jahanshahi et al., 2021). Mokni et al. (2007) showed that resveratrol reduced oxidative stress in the brain of their studied rats. Several studies also showed that catechins (which is the main compound of green tea) have neuroprotective effects and iron chelation activity under *in vitro* and *in vivo* conditions and could reduce the negative effects of Alzheimer’s and Parkinson’s diseases (Mandel et al., 2006, 2008). Some natural products such as curcumin, baicalin, apocynin, and quercetin also have iron chelation activity and neuroprotective effects (Davinelli et al., 2013). Iron is an important factor in the normal activity of neurons, but the accumulation of iron in the brain leads to different diseases (Zecca et al., 2004; Hussien et al., 2018). Iron chelation therapy is considered a new strategy for the treatment of patients with Alzheimer’s and Parkinson’s diseases (Zecca et al., 2004). Iron-related brain diseases may be prevented by early detection of iron accumulation in the brain as well as the use of iron chelators (Zecca et al., 2004). Both hesperidin and coumarin have antioxidant and anti-inflammatory properties. Hesperidin inhibits inflammation by suppressing inflammatory cytokines such as TNF-α and IL-1β, and enroll its antioxidant effect by stimulating the free radical scavenging of antioxidant enzymes SOD and catalase (Li et al., 2015; Kim et al., 2019). On the other hand, coumarin, in addition to its many benefits (e.g., anticoagulant), has neuroprotective properties and stimulates the secretion of acetylcholine at synapses (Hornick et al., 2008; Venugopala et al., 2013). Thus, in addition to beneficial effects such as antioxidant and anti-inflammatory effects, natural compounds often have no serious side effects. Studies show that flavonoid and coumarin compounds could reaction by heavy metal cation, in Figures 8A–C was shown the iron chelation prediction by coumarin and hesperidin (Perron and Brumaghim, 2009; Catapano et al., 2018). According to our results, hesperidin, and coumarin, as two natural compounds, have a high potential in iron chelating. Correspondingly, these compounds cross the blood-brain barrier and chelate excess iron in brain tissue. Further studies on the effects of hesperidin and coumarin on the beta-amyloid formation and iron accumulation in the hippocampus are needed.

## Data Availability Statement

The raw data supporting the conclusions of this article will be made available by the authors, without undue reservation.

## Ethics Statement

The animal study was reviewed and approved by the Ethics Committee of Golestan University of Medical Sciences, Gorgan, Iran (approval numbers: IR.GOUMS.REC.1396.40 and IR.GOUMS.REC.1398.35).

## Author Contributions

MAa carried out the experiment. MJ wrote the first draft of the manuscript and contributed to the interpretation of the results. YS contributed to the interpretation of the results, verified the analytical methods, wrote sections of the manuscript, and edited the manuscript. MAl performed the statistical analysis and designed the figures. MK contributed to conception and design of the study, verified the analytical methods, and supervised the project. All authors contributed to manuscript revision, read, and approved the submitted version.

## Conflict of Interest

The authors declare that the research was conducted in the absence of any commercial or financial relationships that could be construed as a potential conflict of interest.

## Publisher’s Note

All claims expressed in this article are solely those of the authors and do not necessarily represent those of their affiliated organizations, or those of the publisher, the editors and the reviewers. Any product that may be evaluated in this article, or claim that may be made by its manufacturer, is not guaranteed or endorsed by the publisher.
